# Monthly Summary

**Published:** 1894-03

**Authors:** 


### Monthly Summary.

CHLOROFORM NARCOSIS.—What should be done in cases of threatened death from ether or chloroform narcosis? You have seen surgeons under such conditions fill up the patients with whiskey by injection. Prof. Wood, at the International Congress at Berlin, said that he considered this treatment to be little better than murder. Our best surgeons in New York have been in the habit of using whisky under such circumstances, but Wood calls attention to the fact that the patient is in danger of death from narcosis, and alcohol is a narcotic. He prefers a cardiac stimulant which is not a narcotic. The only reason why chloroform is more dangerous than ether is because it does not volatilize so quickly. Ether is quickly eliminated from the lungs, and for that reason a patient has a better chance of getting free from its influence. When your patient is in danger of death from ether or chloroform, what treatment should you adopt? Invert your patient—in other words, have him on his head.

A famous eye doctor of Baltimore, Dr. Chisholm, simply turns the patient upside down and allows no other treatment, and claims he never had a single death from narcosis of any kind. The next thing in point of importance is artificial respiration. The late Dr. Sands told me that the most important thing for a person to do in a case of narcosis was to apply hot cloths over the heart. He thought there was a direct transmission of heat through the chest wall to the heart.

Prof. Wood experimented with a great many drugs, and the only two he approves are strychnine, hypodermically, and the tincture of digitalis. Now for my plan. I have spoken of the great efficacy of the application of hot water cloths over the heart. If that is of value, then how much greater would be the effect produced if you could bring hot water in direct contact with the heart. I think it a much more efficient and prompt proceeding to open a vein and pump in hot salt water to the amount of a pint or quart, then get the effect on the heart of mechanical distension of its chambers. The temperature of the water ought to be 120° F. It has been demonstrated that no albuminoid tissue in the body is coagulated or injured by less than 160° F., and so you are absolutely safe in using this degree of heat.—*Odontographic Journal.*

---

DENTISTS AS READERS.—It has been a constant source of wonder to journalists, who are in position to observe the fact more than others, that dentists care so little for the literature of their profession. It is a lamentable fact that not one dentist in ten is a regular paid-up subscriber to any dental periodical.

Their entire course of reading during the year consists of a few sample copies which drop into their hands from a gratuitous circulation. How any man can be content with this meagre amount of disconnected information concerning the development and progress of his profession, is beyond conjecture.

Fve dollars a year, judiciously expended in subscriptions to dental periodicals, would give any man a course of systematic reading that would not only be wonderfully beneficial by keeping his mind refreshed, but would also keep him posted as to the changes, progress and improvements daily occurring.

Can a man keep posted on the political situation of the day by reading a daily paper once a week? It is just as impossible to keep posted in a profession by reading an occasional sample copy.

Then every dentist needs books of reference. There are times when it would be a pleasure, to look back and obtain *data* on some important event that happened in the distant past beyond the stretch of memory. To provide for this, these volumes could be bound year by year, at a small expense, and the result would be, in a few years, a valuable library would be collected without any appreciable cost.

Every professional man who receives money for his services, and does not keep posted on the improvements of his profession, is taking money not justly due him. If you expect to give full value in service, you owe it to your clientele to keep read and fully posted.—*Editorial in Southern Dental Journal and Luminary.*

---

THE NEW MINERAL CARBORUNDUM.—Incidentally to an attempt to produce diamonds by artifice an American chemist has recently discovered a mineral hitherto unknown—the hardest substance in existence with one exception. It is called "carborundum."

The inventor for making his gems obtained from a concern in Lockport, N. Y., the use of its aluminum smelting apparatus. In reducing that metal electricity is employed, generating an enormously high temperature. As a chance experiment, he put into the furnace a lump of clay together with a piece of graphite, which is pure carbon. The result was some small wine colored crystals of rhomboidal shape. On examination it was found that they were harder than sapphire. Diamond is the hardest of natural minerals; sapphire comes next, and then ruby.

Chemical analysis proved that the crystals were composed of carbon and silicon in a combination hitherto unheard of. It does not occur in nature. The process above described, repeated again and again, produced the wine-colored rhomboids every time. A company has been formed to manufacture them for polishing all sorts of things, even diamonds. They are crushed to powder like emery and made into wheels with a cementing compound. The demand for them is already greater than the supply. At the office of the Geological Survey this new grinding material is to be tried in the preparation of thin slices of stone for microscopical examination. These films of rock—granite, marble or what not—are reduced to such thinness that one can read through them.

Mr. Kunz, the famous expert in gems, believes that most of the precious stones will eventually he produced artificially.

All of them are very simple in their composition—the diamond, for example, is pure carbon; and the ruby is almost pure alumina—and the problem is merely to make their elements crystallize properly. Chemists, who have hitherto confined their attention to taking things apart, are beginning to learn how to put them together again. The English professor, Maskelyne, manufactured diamonds in his laboratory several years ago, though they were too small to have any commerical value. Emeralds have been produced accidently at the pottery works of Sevres, France.—*Phila. Times.*

---

Why do Vulcanite Upper Plates Crack?—By Brevity.—1. Because they are too thin.

2. Because they are too thick.

3. Because they are vulcanized too hard.

4. Because they are vulcanized too soft.

5. Because they are badly articulated.

6. Because the *dens sapientiæ* in the process of growth presses too much on the part of the plate which covers the tuberculosis of the superior maxillary.

7. Because of the want of lower bicuspids and molars.

8. Because they are not worth a cent at any rate.—*Dominion Dental Journal.*

---

The most costly of all metals, save only gallium which is worth $3,000 an ounce, is germanium, which is quoted at $1,125 an ounce. Rhodium is worth $112.50 an ounce; ruthenium, $90 an ounce; iridium $37.50 an ounce; osmium, $26 an ounce, and paltadium $24 an ounce. The last is about equal in value to gold. These metals are of no great commercial value.—*Inventive Age.*

---

Dr. A. W. Harlan Retires.—Dr. A. W. Harlan, editor of the *Dental Review*, has retired from that position.

Dr. Johnson, well and favorably known as a writer, is the new editor of the *Review*.

ANCIENT DENTISTRY.—At the Peabody Museum, at Cambridge, there is a collection of archeological treasures, recently found in Central America, that have special interest to the dental profession. These consist of crania, parts of the skeleton, a collection of teeth curiously filed and inlad, with a fine collection of pottery and instruments which were made from bone, stone, and from a volcanic glass, obsidian, together with carvings and statues. The teeth that were found at Copan, are perhaps more interesting than the skull. Many of these have small circular pieces of green jade inlaid in a cavity that has been drilled by a stone or glass instrument in the face of the incisors or cuspids. These inlays are a little more than an eighth of an inch in diameter, the outer surface is rounded and brightly polished, and as perfectly fitted as it could be by the most skilled operator of to-day, with all the modern instruments at his command. In a few of the teeth the inlays have loosened so that it can be taken out, and there appears to be a white substance, perhaps a cement, between the inlay and the tooth, used to hold the inlay in place. It would seem that this inlay might be some mark of distinction, perhaps used in the mouth of a chief or head man of the people. Some of these teeth are filed and have no inlay. Some are inlaid and not filed. And some are both filed and inlaid. Quite a number of the teeth are badly decayed. Much of this decay appears to be at the cervical border, and there does not appear to be any filling of any kind used to stop decay. None of them were filled for prophylactic purposes. In the teeth from skeleton 8, mound 36, found at Copan, two of the teeth that may have formerly had an inlay were partially filled with something that seemed like a red substance. None of these from this skeleton were filed, but in the lower jaw of the skeleton was found the most interesting curiosity in the whole collection to dentists—a lower, left, lateral incisor that had been carved from some dark stone, and which had been implanted to take the place of one that had been lost. The tartar on it would seem to show that it had been worn for some time during life. This implantation antidates Dr. Younger's experiments by about fifteen hundred years. Many of the teeth were so completely covered with tartar as to form masses nearly double their original size; and in one case an upper molar had the tartar deposited in such a way, and to such a degree, that it formed a shape that articulated on the gum of the lower jaw, where the teeth had previously been lost. In one case, at least, the drilling of the tooth to produce a cavity in which to fit the inlay had encroached on the pulp, and there is distinct evidence of recalcification of pulp tissue at this point.

The whole collection is one of much interest, perhaps the most interesting evidence of prehistoric dental work that is to be found in any museum, and is well worth a visit to Cambridge to see. They are suggestive of what the prehistoric inhabitants of this hemisphere were.—R. R. Andrews in *International.*

---

DENTISTS IN POLITICS.—In our last issue we stated that one of our confreres sits in the Ontario Parliament. We have received seven notifications that the statement is incorrect, that in the bye-election he was defeated. We regret this exceedingly, both for the sake of the electors and the profession.

We would just observe, *en passant*, that we wish there was the same anxiety to supply us with correct news, as to correct our news. These personal items are interesting, and we should receive them by the dozen every month.—*Dominion Dental Journal.*

---

IF you can successfully save a tooth with amalgam or anything else, the pay should be commensurate with the services rendered, be it two, three, five or ten dollars. No wonder we hear the cry of "down with amalgam!" especially when we find it manipulated by the hand of carelessness or ignorance. It is in poor company and utterly helpless.—E. G. Clark.

